# Scaffolding medical student knowledge and skills: team-based learning (TBL) and case-based learning (CBL)

**DOI:** 10.1186/s12909-021-02638-3

**Published:** 2021-04-26

**Authors:** Annette Burgess, Elie Matar, Chris Roberts, Inam Haq, Lucy Wynter, Julian Singer, Eszter Kalman, Jane Bleasel

**Affiliations:** 1 Faculty of Medicine and Health, Sydney Medical School - Education Office, The University of Sydney, Sydney, NSW 2006 Australia; 2 Faculty of Medicine and Health, Sydney Health Professions Education Research Network, The University of Sydney, Sydney, Australia; 3 Faculty of Medicine and Health, Sydney Medical School – Central, The University of Sydney, Sydney, Australia; 4 Faculty of Medicine and Health, The University of Sydney, Sydney, Australia

**Keywords:** Case-based learning, Team-based learning, Medical education, Small group learning

## Abstract

**Background:**

Two established small-group learning paradigms in medical education include Case-based learning (CBL) and Team-based learning (TBL). Characteristics common to both pedagogies include the use of an authentic clinical case, active small-group learning, activation of existing knowledge and application of newly acquired knowledge. However, there are also variances between the two teaching methods, and a paucity of studies that consider how these approaches fit with curriculum design principles. In this paper we explore student and facilitator perceptions of the two teaching methods within a medical curriculum, using Experience based learning (ExBL) as a conceptual lens.

**Methods:**

A total of 34/255 (13%) Year 2 medical students completed four CBLs during the 2019 Renal and Urology teaching block, concurrent to their usual curriculum activities, which included weekly TBLs. Questionnaires were distributed to all students (*n* = 34) and CBL facilitators (*n* = 13). In addition, all students were invited to attend focus groups. Data were analysed using descriptive statistics and thematic analysis.

**Results:**

In total, 23/34 (71%) of students and 11/13 (85%) of facilitators completed the questionnaires. Twelve students (35%) participated in focus groups. Findings indicate their experience in CBL to be positive, with many favourable aspects that built on and complemented their TBL experience that provided an emphasis on the basic sciences. The learning environment was enriched by the CBL framework that allowed application of knowledge to solve clinical problems within the small groups with consistent facilitator guidance and feedback, their capacity to focus discussion, and associated efficiencies in learning.

**Conclusion:**

While the TBL model was integral in developing students’ knowledge and understanding of basic science concepts, the CBL model was integral in developing students’ clinical reasoning skills. The strengths of CBL relative to TBL included the development of authentic clinical reasoning skills and guided facilitation of small group discussion. Our findings suggest that delivery of a medical curriculum may be enhanced through increased vertical integration, applying TBL in earlier phases of the medical program where the focus is on basic science principles, with CBL becoming more relevant as students move towards clinical immersion.

## Background

Contemporary medical education employs a variety of small group learning methods [1–3]. Two such established small-group learning paradigms in medical education include Case-based learning (CBL) and Team-based learning (TBL). Both methods involve inquiry and are predominantly implemented in the early years of the curriculum [2, 4, 5]. Characteristics common to the pedagogy of both CBL and TBL include the use of an authentic clinical case, active small-group learning, activation of existing knowledge and application of newly acquired knowledge [2, 6, 4, 7]. However, there are also significant theoretical and practical variances between the two teaching methods, and a paucity of studies that consider their similarities and differences, and how both approaches fit with curriculum design principles. In this paper, we explore the theoretical and practical differences between CBL and TBL by drawing on students’ and facilitators’ perceptions of the two teaching methods within a medical curriculum.

### Case-based learning (CBL)

CBL is a method of teaching used across many disciplines and designed to promote students’ development of analytical thinking skills and reflective judgement, by engaging them in discussion about complex, real life clinical scenarios [4]. In CBLs, students are encouraged (in groups of six to 10) to engage in peer learning and apply new knowledge to these authentic clinical problems under the guidance of a facilitator. As with TBL, the facilitator is ideally a content expert, and is encouraged to correct, redirect and provide feedback to students [7]. In this way, CBL fits between structured and guided learning [4], and provides the benefit of developing clinical problem-solving skills at an early stage of training [8]. Structure is provided by the case presented, and the unfolding questions that follow with a timing guide. Through careful questioning and by role modelling their own clinical reasoning, facilitators encourage students to develop their skills in analytical thinking and reflective judgement in preparation for clinical practice. When compared to problem based learning (PBL) and TBL, CBL is less time consuming, because the facilitator draws students’ focus to key points of the clinical case [7]. Indeed, while a CBL session often takes a similar trajectory to what is encountered in the real world in the exploration of a clinical case (formulation of differential diagnoses; proposal and interpretation of relevant investigations; generation of treatment plans) it can equally be modified to focus discussion on any one of these aspects of the case. Thus, the CBL format is versatile and can be incorporated to suit a range of time-restricted settings. CBL encourages a structured and critical approach to clinical problem-solving, and, in contrast to PBL, is designed to allow the facilitator to correct and redirect students [7].

### Team-based learning (TBL)

TBL offers a student-centred, instructional approach for large classes of students who are divided into small teams of typically five to seven students to solve clinically relevant problems [2, 9]. As the name suggests, the focus of TBL is the learning that takes place within and between different teams. As such, TBL includes elements favourable to preparing students to work in teams, synthesis information, and communicate with each other [10]. This results in the highly structured format of TBL relative to CBL, which includes a preparatory (pre-reading), readiness assurance, feedback and problem-solving phase [7]. A distinguishing feature of TBL is the ‘readiness assurance’ phase which makes use of repeated testing, requiring an individual test and team test, followed by provision of immediate feedback and clarification. A key logistical difference relates to the high student-teacher ratio format of TBL, which permits one content expert to effectively facilitate a large number of teams (for example, 12 groups of 6 students in one classroom).

While reported student feedback on both CBL and TBL is generally positive [2, 4, 7, 11], there are few studies reporting on how these pedagogies are vertically applied in medical education curricula, or studies contemplating how they may co-exist within a curriculum to improve the student experience, and how both approaches fit with current instructional design principles. Current literature provides only comparative studies between CBL and TBL*,* whereas this current study considers how the two pedagogies may be integrated within the curriculum. A study by Koles and colleagues (2005), comparing case-based learning group discussion with TBL in year 2 medical students, found no difference in student satisfaction with either method, although students indicated the smaller groups of TBL (5, 6) compared to CBL (20) enhanced the peer learning process [12]. Additionally, results showed no difference in examination scores between the student groups, although students in the lowest academic quartile demonstrated less deterioration of knowledge after TBL. Gonzalo and colleagues (2018) compared Year 1 medical student examination performance following participation in a modified CBL, where students were assessed by their facilitator on non-cognitive skills using a subjective five-point Likert scale [13]. Their findings indicated that TBL Individual Readiness Assurance Test (IRAT) scores provided a strong prediction for final examination scores, but CBL scores did not.

### Research context

The study was carried out in 2019 at Sydney Medical School, in Year 2 of the four-year graduate entry Sydney Medical Program (SMP). In 2017, TBL was successfully introduced to Years 1 and 2 of the Sydney Medical Program (SMP), as a replacement for Problem-based learning (PBL). The TBL and PBL models have been previously reported [3]. TBL is used to teach integration of basic sciences and clinical concepts in the first 2 years of the medical program. In preparation for a new SMP commencing in 2020, where Year 2 student teaching will predominantly shift from university to hospital-based teaching, with earlier clinical exposure, CBL was piloted in 2019 in parallel to existing TBL sessions in the curriculum. This provided a unique opportunity to study students’ perceptions of CBL and draw comparisons with their experience of their existing Year 2 TBL sessions. In this context, CBL was piloted in Year 2 of the medical program. Here, we report on a pilot implementation of CBL at three Hospital based Clinical Schools in Year 2 of a graduate entry medical program during the 2019 Renal and Urology teaching block.

### Experience based learning (ExBL) framework

Theoretical frameworks offer valuable lenses to help us understand and conceptualise participants’ perceptions of their teaching and learning experiences. The ExBL model developed by Dornan and colleagues (2014) suggests that learning objectives of medical students are achieved through joint participation in authentic activities, where the existing resources are used to construct the optimal conditions for learning [14]. The ExBL model suggests that student learning is fostered through three key areas of support:

1. Organisational: the learning activity needs to sit appropriately within the curriculum, with opportunities to actively participate.
2. Pedagogic: during the learning activity, teachers, mentors and role models need to be supportive.
3. Affective: an inclusive and warm environment should be provided during the learning activity.

Using the ExBL framework to interpret our findings, the purpose of our study was to explore students’ and facilitators’ perceptions of CBL, drawing some comparisons with their experiences of TBL. Our specific research questions were:

- How do the CBL and TBL models align with the medical curriculum and assist in achievement of student learning outcomes?
- How do the key features of CBL and TBL support learners during implementation?
- What are the perceived strengths and limitations of the learning environments created when using the CBL and TBL models?

## Methods

### CBL design

Four CBL sessions (CBLs) were run on a weekly basis during the renal and urology teaching block, concurrent to the usual curriculum activities, which included weekly TBL sessions (TBLs).

### Content of the CBL sessions

The weekly learning topics, outlined in Table 1, were aligned to complement the TBL case of each week. The CBLs were designed to develop students’ capabilities in clinical reasoning, assessment, diagnosis and management of patients with these conditions.

**Table 1:** Weekly learning topics of Renal and Urology teaching block

CBL CASE	DISEASE/CONDITION	CASE TITLE
1	IgA Nephropathy	“Murky waters”
2	Acute Kidney Injury	“I just don’t feel hungry”
3	Hypertension	“A bad headache”
4	End Stage Kidney Disease	“A dragging Pain”

### Structure of the CBL sessions

Year 2 medical students were allocated to their CBL ‘teams’, each consisting of five or six students. Each CBL session took place in a small room to accommodate individual groups and each CBL session was 1.5 h in duration. All students were expected to actively participate in the CBL session, and the facilitator was expected to take an active role in guiding discussion and student learning. The steps in the CBL were 1) presentation of the clinical problem 2) seeking out extra information (undertaken during class) 3) discussion with the facilitator. Although prior student reading is usually part of the process in CBL, this was not included, since it was anticipated that the lectures and learning activities of each week would prepare students for the CBLs.

#### Facilitation

A total of 13 clinicians based within the different clinical schools participated as facilitators for the CBLs. The allocation of facilitators varied at each clinical school in terms of expertise, ranging from senior medical registrar to nephrology consultant. Facilitators were provided with a 30 min training session, a facilitator guide, and the cases 1 week prior to each CBL tutorial.

### Structure of team-based learning

Students continued to attend their usual TBL sessions. The TBL sessions were held once per week for 2.5 h (including 40 min for the Readiness Assurance Process, and 1 h, 45 min for application of clinical problem-solving activities). Approximately 50 students were allocated to each TBL class. Five to six students were allocated to each small group within these classes. Each TBL class had three discipline expert facilitators: one renal consultant, one basic scientist and one medical registrar. The TBL method consisted of pre-class preparation, IRAT, team readiness assurance test (TRAT), feedback, and clinical problem solving activities as previously reported [3]. Both TBL and CBL were presented to the students using the Learning Management System (LMS) (kuraCloud), which would unfold the case as it progressed, and provide relevant images and videos.

### Study design

#### Sampling and participants

A total of 34/255 (13%) Year 2 medical students completed four CBL sessions (CBLs). This was undertaken concurrently to their usual curriculum activities, which included weekly TBL sessions (TBLs). Students were sampled from three Clinical Schools based at metropolitan teaching hospitals. Sampling of students at two Clinical Schools was purposive, that is, students were selected based on their availability according to their timetable.

### Data collection and analysis

#### Questionnaires

Quantitative and qualitative data were collected from students and facilitators by questionnaire. A separate questionnaire was designed for students and facilitators. The questionnaires included closed items, (using five point Likert-scale, with 1 being ‘strongly disagree’, and 5 being ‘strongly agree’). The questionnaire items were based on the questionnaire used in our previous study [3], and were aligned with the selected theoretical framework. They were based on student learning outcomes; on the quality of team processes [15]; and the qualities of the learning environment, such as tutor interactions. Each survey included 15 closed items. Open-ended questions were also asked to elicit a deeper understanding of the most positive aspects of participating in the CBLs, how the CBLs could be improved, and how they compared and built on the TBLs and medical curriculum.

Questionnaires were distributed to all student participants (*n* = 34) and all CBL facilitators (*n* = 13) following completion of all four CBLs. Quantitative data were analysed using descriptive statistics. A thematic analysis of the qualitative data was performed within each category [16]. A portion of the data was read by the first author and analysed to identify initial themes. Following negotiation of meaning with the second author, a coding framework was developed and applied to the full set by the first, second and third authors.

### Focus groups

To gain a deeper understanding of students’ perspectives and experience of the CBL and TBL sessions, all students were invited to attend one of two focus groups. The focus group questions were semi-structured, and aligned with the questionnaire items and theoretical framework. The focus groups were recorded and transcribed verbatim. Following consultation with the second and third authors, the first author (AB) used framework analysis to code the focus group data, using ExBL as a conceptual framework.

### Ethics approval

The University of Sydney Human Research Ethics Committee approved the study.

## Results

### Student questionnaire responses to closed items

In total, 23/ 34 (71%) of participants completed the questionnaire regarding their CBL experience.

**Fig. 1:**
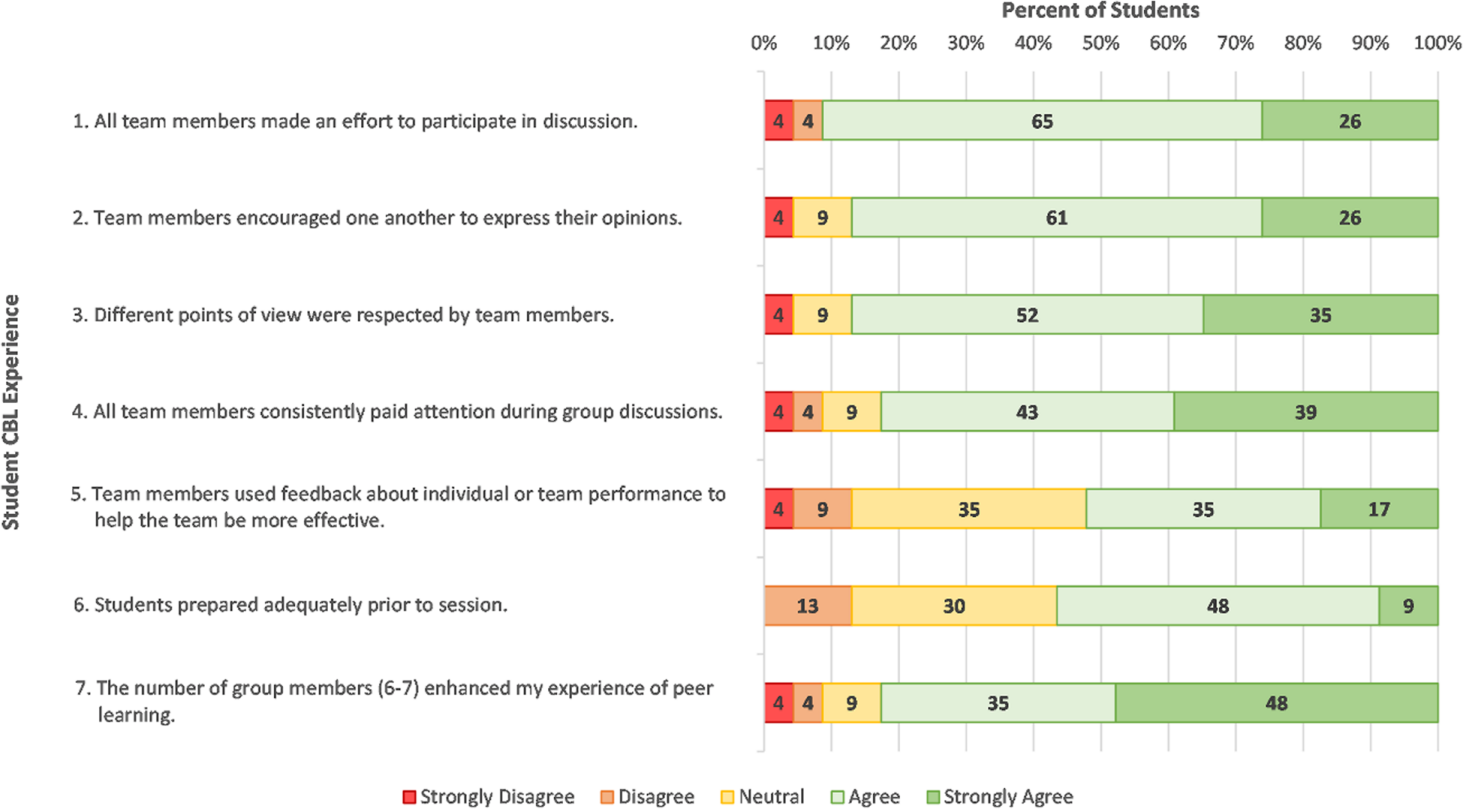
Student responses to closed items regarding their experience in CBL items 1–7 (*N* = 23)

Student responses relating to team dynamics are displayed in Fig. 1. Most students agreed that team members made an effort to participate, encourage, listen to others, utilised feedback, and were adequately prepared.

**Fig. 2:**
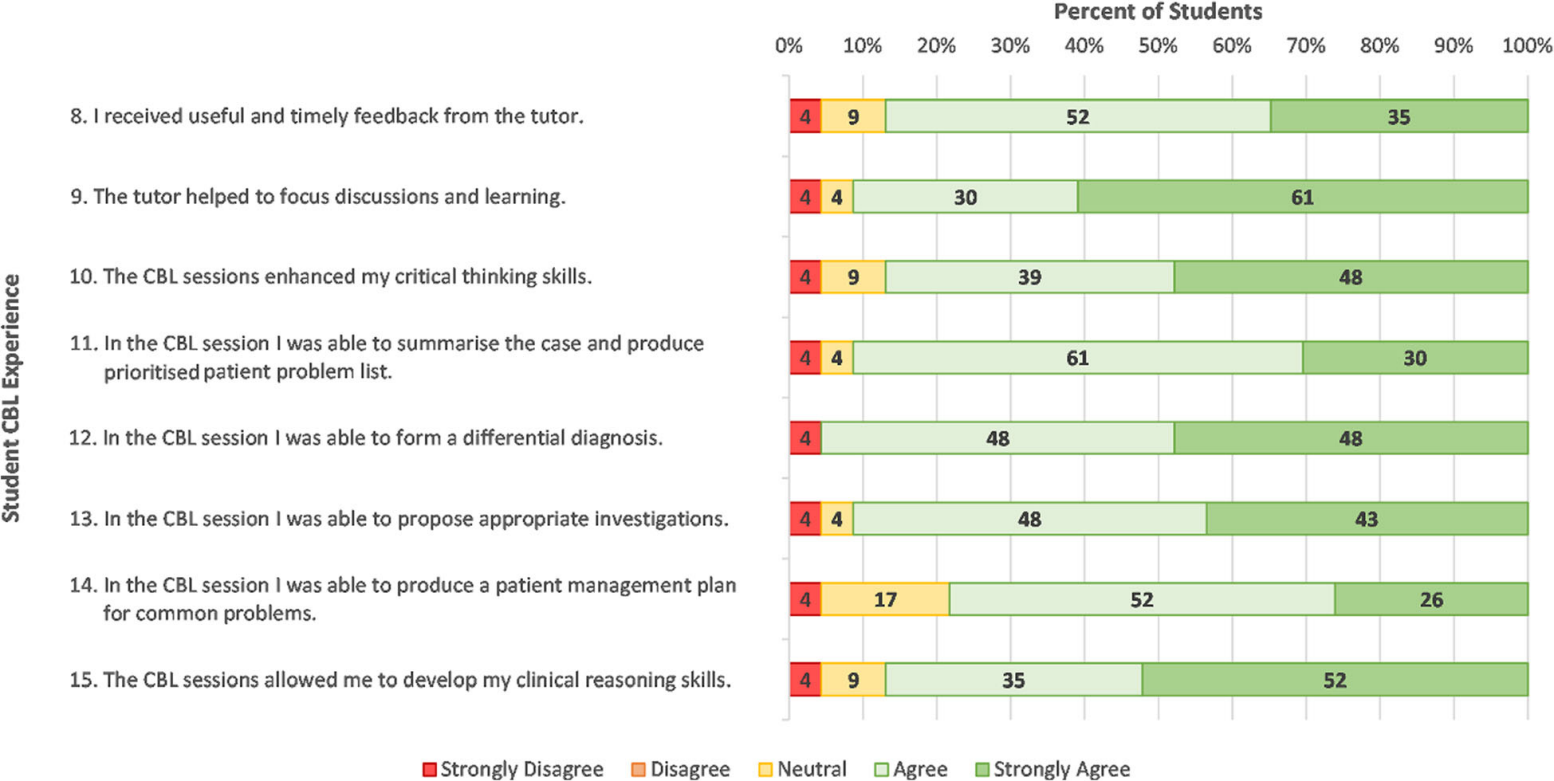
Student responses to closed items regarding facilitator interactions and learning outcomes (items 8–15) (*N* = 23)

Student responses to items regarding interactions with the facilitator and student learning outcomes are displayed in Fig. 2. Most students felt the facilitator provided *“useful and timely feedback”* (item 8) and *“helped to focus the discussion and learning”* (item 9). Most students felt they were able to summarise the case and produce a prioritised problem list, form a differential diagnoses, and propose appropriate investigations. Students were less confident (78% in agreement) in their ability to produce a patient management plan for common problems (item 14). Notably, most (87%) of students agreed that the CBL session enhanced their critical thinking skills and clinical reasoning skills (items 10 and 15).

### Facilitator questionnaire responses to closed items

In total, 11/13 (85%) of facilitators completed the questionnaire regarding their CBL experience.

**Fig. 3:**
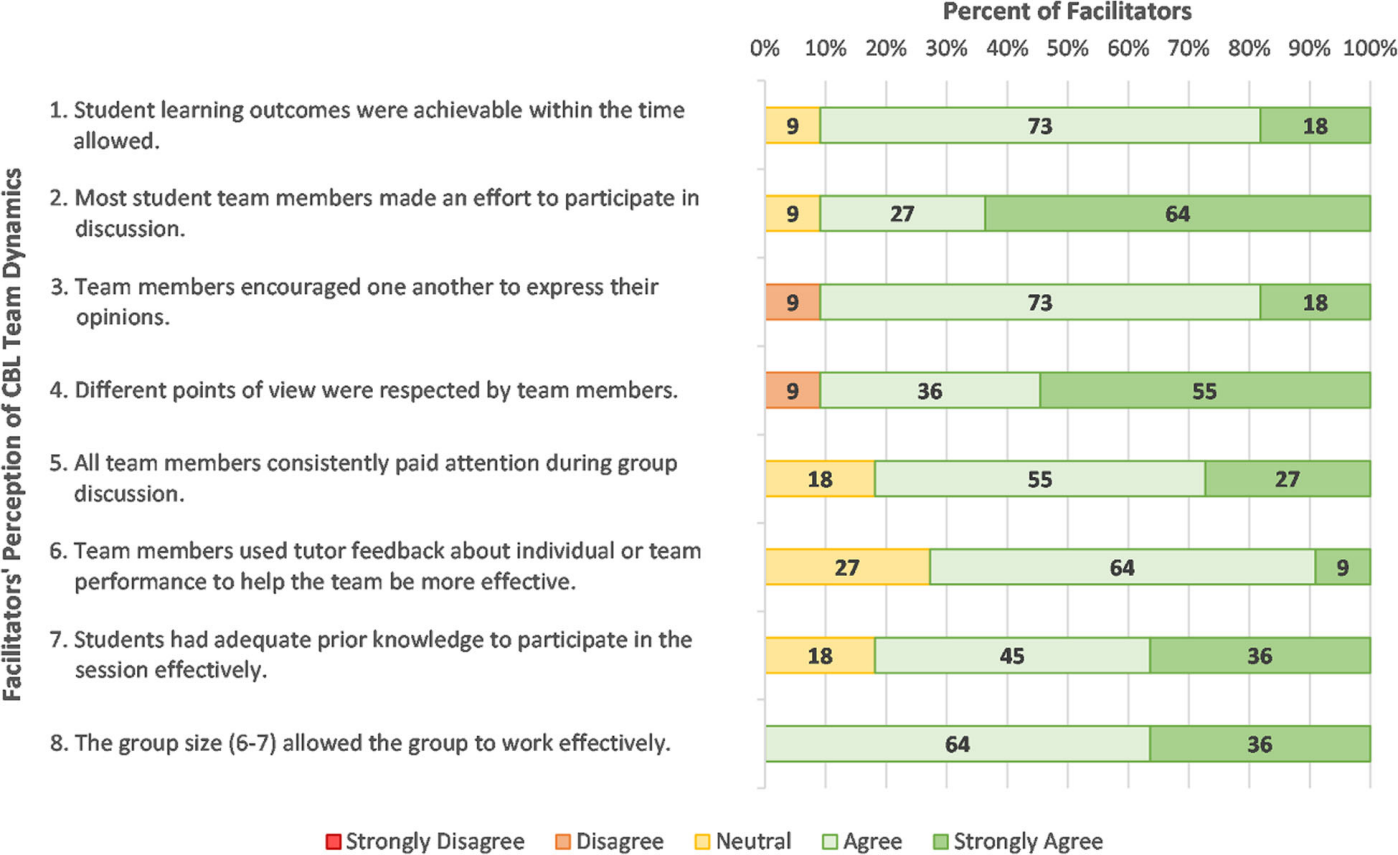
Facilitator responses to closed items regarding students’ team dynamics in CBL (items 1–8) (*N* = 11)

Facilitator responses to closed items regarding student team dynamics are displayed in Fig. 3. Importantly most facilitators (91%) felt the learning outcomes were achievable within the CBL timeframe (item 1). Responses indicate that students had adequate prior knowledge, participated in discussion, and encouraged the participation of their peers. However, there was less agreement (73%) that students utilised facilitator feedback (item 6).

**Fig. 4:**
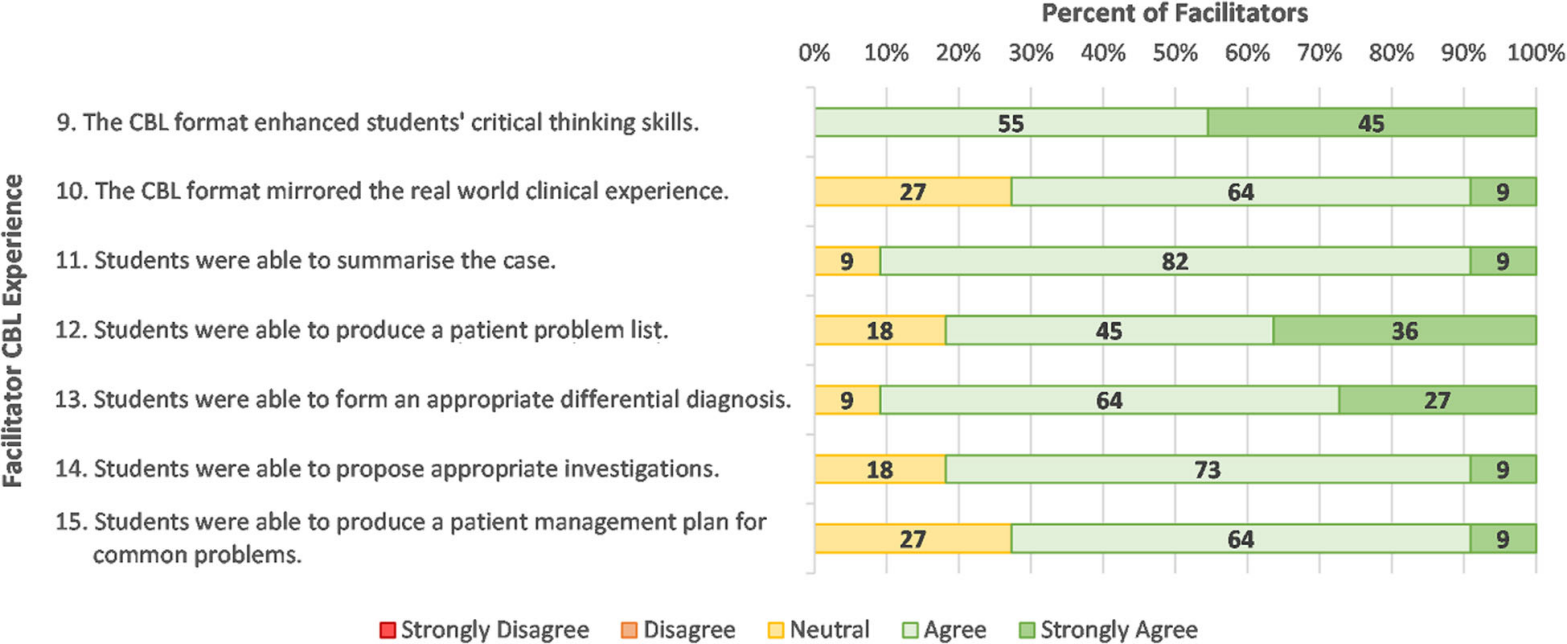
Facilitator responses to closed items regarding their experience in CBL items 9–15 (*N* = 11)

Facilitator responses regarding development of students’ clinical reasoning skills are shown in Fig. 4. Almost all (95%) facilitators agreed that the CBL session enhanced their critical thinking skills and clinical reasoning skills. Most facilitators felt that students were able to summarise the case, produce a prioritised problem list, form an appropriate differential diagnoses, and propose appropriate investigations. In line with students’ own responses, the facilitators were less confident (73% in agreement) in students’ ability to produce a patient management plan for common problems (item 15).

### Responses to open-ended questions

#### Best features of CBL

Student and facilitator responses to open ended questions regarding students’ perceived best features of CBL are shown in Table 2. Both students and facilitators felt the CBLs mirrored the format of a clinical consultation and encouraged critical thinking, with systematic application of knowledge. Students found this format allowed them to identify knowledge gaps. They found working in small groups with one facilitator more conducive to learning and participation than the large classes of TBL, and valued the continued guidance and feedback from a clinical expert in a small group setting. Both students and facilitators reported the clinical aspects of the case and shorter duration of CBLs helped to focus their learning. Compared to the large classes of TBL, facilitators found the small group size of CBL encouraged interactive learning with students more forthcoming with asking and answering questions. They also reported they could readily role model their clinical reasoning to impart their knowledge.

**Table 2:** Student responses to “the best features of CBLs?” and facilitator responses to “the most positive aspects of tutoring CBLs”

Theme	Example of students comments
***Student responses to “the best features of CBLs”***	
Students felt the session mirrored the format of a clinical consultation.	*I liked how the sessions were set up as if it was a clinical consultation. I liked how systematic it was.* *Really help us in forming Differential Diagnoses.*
Opportunity to speak directly with clinicians and receive immediate feedback.	*Ability to speak to clinicians directly, highly interactive.* *It was good to receive immediate feedback and answers from tutors. For example, there could be ongoing conversations between tutors and students about the case without interruptions, unlike TBLs. The flow and pace of CBLs were good.*
The faster pace of CBL (1.5 h) helped students to stay focussed and engaged.	*The structure and shorter time required for this activity were so much better than TBL. It is more clinical based than the TBL, fast paced enough that people stay engaged. We were able to get to the point of the case and learn more about the clinical features and management of the condition rather than going more in depth behind the science and brushing over the clinical information.*
The smaller group size gave students more confidence to ask the facilitator questions, and receive immediate responses.	*The small group size made CBL so much more effective than any TBL we have done thus far. Being able to discuss directly with the tutor made learning much more pointed and efficient. I also felt much more confident to ask questions than I do in TBL because of the group size.* *It was a small learning environment, - great one to one teaching. Questions are able to be answered right away without feeling pressure.*
CBLs were more relevant for future practice, although students recognised the need for basic science in TBLs.	*Small groups were very beneficial. That combined with the relatively minimal guidance given in the CBL material meant that we had great discussions with the doctors. I felt it was more relevant for future practice, however, I appreciate the need to balance with basic science in the earlier years of medical degree - There was less basic science learning than in the current TBL.*
The process of CBL helped students to identify gaps in their knowledge and focus on the clinical aspects of the case.	*We would go through the case first alone and discuss as a group. Then the tutors would jump in if we were stuck. We get to know more about the clinical side of the disease* e.g. *what are the more common presentations of a disease, what test would clinicians prefer doing and what are the diagnostic and treatment algorithms. The part where we were asked about what extra information we wanted to look up is good. This helps us identify gaps in our knowledge and to practice our EBM skills.*
***Facilitator responses to “The most positive aspects of tutoring CBLs”***	
Facilitators felt that having the cases based on real life patient cases encouraged students’ critical thinking and systematic application of knowledge to a problem.	*Encouraged critical thinking and allowed for good group contribution and joint learning. Mirrored real life situation as closely as possible.* *Interactive, more close to a real life meeting, good positive interaction encourage students to use knowledge they have and organise it/apply it systematically to a clinical proble*m.
Facilitators found working through the case simulated real life patient presentations The smaller student groups of CBL, compared to TBL, encouraged group discussion.	*I think it is a great way to move through a patient case and it encourages contribution. I believe it closely simulates a real life presentation quite well but goes through it at a good pace to encourage participation and teamwork.* *Students were interactive and very keen to learn (compared to a larger group setting* e.g. *TBL). The case was interesting and seem to be at a level that was appropriate for students. The interactive nature of the material made the session more interesting.*
Facilitators found the CBLs to be more interactive than TBLs, and students were more forthcoming in answering questions.	*Provides interactive learning environment, more reflective of real clinical scenarios. Engaged students who were forthcoming in attempting answers.* *Good student engagement. The students were quite knowledgeable and very receptive. All were willing to interact so it was not difficult to elicit responses. CBL was effective at getting them to think critically about a case.*
Facilitators had a greater opportunity to role model their clinical reasoning, and impart their knowledge compared to TBL.	*Students found that it was a lot more clinically relevant than TBLs and easy to understand. Lots of opportunities for tutors to distil clinically useful knowledge.* *Interesting cases Information revealed bit by bit, which helped to formulate the differential and provisional diagnosis.*

### Suggestions for improvements to CBL

Student and facilitator responses to open ended questions regarding the areas for improvement of CBL are illustrated in Table 3. Facilitators felt the objectives of each CBL session needed to be more explicit, and requested timing guides to ensure they moved through each session at an appropriate pace. Some students reported variability in the training and experience of their CBL facilitators, ranging from medical registrars to consultant nephrologists, indicating they appreciated the availability of consultants in TBLs. Students commented that the CBL session would benefit from assigned pre-reading preparation, as occurs with TBL. Both staff and students found excessive repetition in some of the questioning formatted within the CBLs. They also reported flaws in the online platform through which material was presented, and in particular, the videos, did not always work; and felt more images and investigations would be helpful to reach decisions on diagnosis and management. Some facilitators suggested having MCQs at the end of the session to solidify student learning.

**Table 3:** Student and facilitator responses: “Most difficult features of CBLs and suggestions for improvement”

Theme	Example of student comment
***Student responses: most difficult features of CBLs and suggestions for improvement***	
Students felt it would be beneficial to have tutors who were specialists in their fields, as occurs in TBLs.	*The tutors were great, however most of them did not come from the field of nephrology, and were hence not able to answer all our questions.* *What is better about the TBLs, is that we usually have at least one specialist (consultant) and a physiologist present. This means that they are truly experts in their field, and any question related to the field can be answered.*
Students would prefer that less information was provided to them on the CBL case.	*Would be nice if not all cases were so clear cut from the beginning, adding distractors may make it more realistic.* *The title of CBL and TBL both sometimes will give away the diagnosis. It would be less helpful / fun if students already go in with an idea in mind.*
Students felt there were a lot of repetitive questions that should not have been repeated.	*Removing the repetitive questions such as “what are the patient problems” as asking this 3 times we did not add any new information and felt as though we had already discussed everything to be discussed with the tutor.*
Students recommended the inclusion of pre-reading, as occurs for each of the TBLs.	*Having some pre work or some reading about the case as whilst we had some lectures on the context of the cases, clinically we did not have that much knowledge so it was hard to know the best ways to manage or the specific treatments without having learnt it before.*
The online technology was not always working and students felt additional images would be helpful.	*Some of the videos didn’t work so we felt as though we were missing key bits of clinical information that would help us progress with the case. We felt that at some times we were not given enough information in order to make diagnostic or management decisions.* *I think more images (medical imaging, histo etc) and explanation about the pathophys of the underlying condition would be good*
***Facilitator responses: most difficult features of CBL and suggestions for improvement***	
Tutors found timing segments of the session difficult and would like timing schedules. They found at times, the tutorials were too fast paced.	*Timing - would be nice to get some time-guides throughout the resource to know we are on track.* *Time frame was very tight. I felt I had to keep the session moving relatively quickly and at times perhaps cut the team off early rather than giving them more time to discuss the issues*
Tutors would like the objectives for the session to be emphasised to ensure the session remains focussed.	*Clear learning objectives for the session (I may have missed this) - because sometimes discussions can get slightly side tracked if we are not careful.* *Staying on track, navigating the case. As a tutor it is hard to know how much information I am able to volunteer or whether I should encourage them to research it themselves.*
Tutors found the questions within the CBL to be repetitive at time.	*I felt some parts were fairly repetitive, constantly asking for lists of problems and so forth, I think it could be clearer what the students were expected to contribute as they moved through.* *Less repetitive questions - especially questions like what are the issues was asked multiple times*
Tutors felt the questions needed refining, and to have more practical clinical applications.	*The EBM question was a bit out of place. I think this was a big deviation from the clinical process but I understand its role. Perhaps this question needs to be softened, and more angled about seeking knowledge about management,* etc. *For example, a dilemma about co-prescribing medications, or nephrotoxicity and needing to check MIMs might have been worthwhile. Similarly, guides on how to manage Warfarin dosing,* etc. *Just a little more practical.*
Tutors four delivery of CBL on Kuracloud to be distracting, and some indicated they would prefer paper cases.	*I find the technology can be a bit distracting, students engaged better when we discussed the answers rather than pressing the professor button.* *There were flaws in the online platform with some incorrect answer on particular sessions (i.e the answer for one section was repeated on 2 other sections instead of their respective answers.*
Tutors felt more images would be helpful.	*More images, blood and investigations to interpret “good overall minimal improvements need to be made perhaps a brief review (5 min) of anatomy/physiology of relevant system prior to clinical scenario”.*
MCQs at the end of the session as a way to solidify knowledge.	Some MCQs after to solidify concepts.

### How CBL builds on and compares to the TBL experience

Student and facilitator responses to how CBL built on and compared to the TBL experience in the medical program are shown in Table 4. Students and facilitators felt the CBL sessions provided an opportunity to clarify concepts introduced at the TBL sessions, and apply their basic science knowledge, with more opportunities to be actively involved. They felt the smaller groups and constant presence of a facilitator in CBL fostered greater peer discussion and learning compared to TBL. Both facilitators and students felt the CBLs provided an opportunity to synthesise knowledge acquired in the clinical setting, and gain valuable insights in patient investigations and treatments. Facilitators felt the CBL session provided an efficient and effective means to integrate student learning from lectures and tutorials, and build on the TBLs by allowing students to apply their basic science knowledge to develop their clinical reasoning, history taking and examination skills. Both facilitators and students commented on the time efficiencies of CBL compared to TBL.

**Table 4:** Student and facilitator responses: “How did CBL build on and compare to the TBL experience?”

Theme	Example of student comments
**Student responses: How did CBL build on and compare to your TBL experience?”**	
Consolidated knowledge learnt in the TBL and increased students’ understanding of clinical concepts.	*It helped to consolidate a lot of the knowledge I got from TBL and added on to it.* *The whole structure made more sense and the clinical picture was followed through continuously from beginning to end. It gave me a more clinical understanding of the renal conditions that I could not grasp from TBL sessions.*
CBLs helped to synthesise learning students have gained in the clinical setting. Students valued gaining insights in different investigations and treatments, and relevant clinical symptoms.	*Having CBLs right after clinical sessions kept things fresh and was a good way to synthesize what we learned during tutorials.* *It helped to expose me to different investigations and treatments I did not know about. It also helped me to pick up on relevant clinical symptoms more easily.*
CBL was more focussed, practical knowledge relevant to clinical placements.	*The shorter time allocation meant we were more focused and felt I was able to maintain focus for the whole session.* *The CBL used a lot of the same skills, but was more focused, which was better. More practically useful knowledge,* i.e. *what is likely pathology given presentation and what tests/exams/Hx to do to rule out DDx*
Participants felt that CBL provided an opportunity to clarify any misconceptions that had arisen during the TBL, with the smaller group size ensuring immediate feedback from the facilitator.	*CBL provided a good opportunity to clarify concepts introduced at TBL. It is a better environment to ask questions. Much more opportunity to be actively involved!* *Having a smaller group size with individualised tutors means all questions could be asked on the spot which was a great experience.*
Students found the CBLs provided a greater clinical focus, and allowed them to apply the basic sciences.	*I thought it was good that it was more clinically focussed - a good practice at taking the basic sciences and applying it.* *It required me to think more critically about the case compared to TBL. Much better in forming differentials since we do not hang on the weekly topic and narrow down our diagnoses*
Since the main focus of TBL is on the basic science, students found the CBL provided greater opportunities to discuss the clinical aspects of the patient case, such as clinical diagnosis and management.	*CBL is good to view medical conditions with another lens as TBL mainly focuses on basic science and pathophysiology. CBL supplements well with the discussion on clinical diagnosis and management.* *It provided an experience to discuss a clinical perspective for different conditions which I feel is beneficial as a way to develop a more concrete understanding of more pathologies. I do not feel it is a replacement for TBL though as the BCS [basic clinical sciences] focus of TBLs is definitely of value in its own way.*
Students found TBL provided greater time efficiencies and focussed the clinical discussion.	*I preferred the CBL experience It was shorter, more clinical based and the group size was more intimate and felt it fostered learning of the clinical aspects of cases which we don’t get as much in lecture learning or in TBLs.* *CBL is a better, more efficient, and yet more comprehensive way to teach and foster group work for the medical program.*
Some students commented that TBL and CBL each brought their own benefits. CBLs allowed for greater in depth discussion, and TBL provided a structured format to work through the case.	*I enjoyed both CBLs and TBLs for different reasons. CBLs due to the smaller group size and more personal discussions. TBLs for the better formatting/structure of the case on kuraCloud*. *I think CBLs and TBLs went hand in hand and improved my understanding in both sessions.*
Increased facilitator contact, with greater opportunities to ask questions and feedback.	*I like CBL better because you can ask more questions. It was far better. The shorter amount of time and the more one to one teaching was amazing.* *Compared to TBL, CBL is more “personal” as we get two tutors per group. It is a better environment to stimulate discussion.*
Students valued the constant presence of the tutor with their group and the provision of guided learning from experienced tutors throughout the CBL.	*Much better than tbls, smaller groups with tutors the whole way through guiding you was better than tutors floating between groups.* *More clinic and more efficient than TBLs because of the better tutor to student ratio. It was so much better, I learnt so much more in this setting.*
**Facilitator responses: How does the CBL build upon student’s experience in the medical program?**	
Facilitators felt the CBL sessions built on the students’ TBLs by requiring students to apply their basic science knowledge to develop their clinical reasoning, history taking and examination skills.	*It is a good culmination of relevant skills that mimic a real life situation, requiring students to apply basic science, clinical reasoning and history and examination skills.* *I think it adds clinical perspective to material covered in other parts of the program* e.g. *TBL, lectures and bedside teaching. The CBL seem to tie in clinical skills* e.g. *examination, history taking with the underlying science/pathophysiology and clinical reasoning.*
Tutors felt CBL gave students the opportunity to apply their knowledge to a clinical case.	*It provides an opportunity to incorporate the knowledge students learnt in lectures and clinical experience from bedside tutorials into a clinical case therefore integrates the learning from the other teaching they have in the program.*
Through CBLs, students were able to relate the case to patients they had seen on the wards during the week.	*Students were able to relate the cases to patients that they had seen on the wards.*
Tutors felt there were time efficiencies to CBL.	*Team based, interactive I believe it fits in well and is an effective use of time at this stage of their skills / knowledge, enhances learners experience. It fits well.*

#### Focus group results

In total, 12/34 (35%) of participants attended a focus group session. Of the participants, seven were female and five were male. The focus group sessions were each 1 h in duration, conducted by the first author (AB). The results are presented below using the conceptual framework of ExBL [14].

#### Organisational support

Students felt the CBL session **fitted well with the remainder of their teaching week**:

*You have a case in front of you, and just, kind of, go through it step by step. So then I felt that I quite enjoyed having that sort of framework in mind, and then learning the specifics of it throughout the rest of the week*

They also indicated the **TBL and CBL models helped to spiral their learning**:

*By pulling on parts of my degree and more parts of my knowledge that we’ve learnt, rather than just being, we’re in renal, this is the little block that we’re pulling our information from which I thought was really helpful because I know that I went and reviewed a few other things from, you know, previous years.*

Students found the **CBL sessions to be more clinically relevant and focused than TBLs**:

*I think I learnt way more l than I do in TBL because it’s like very clinically-based, and it’s more focused on management and treatment and stuff which we don’t get to do really in TBL - it was way practical oriented. Especially because the tutors were all clinicians, so they were always talking about what wouldn’t be feasible on the ward – I really enjoyed that.*

Students found **TBL valuable in preparing for written examinations, and CBL in preparing for clinical placements:**

*CBL makes us think in a more in a critical way about what we would do in a clinical context, and especially going into third year, it’s really useful to start building that kind of thought process. TBL just gives it to you and, it’s more about the theory, which is great for exams.*

### Pedagogic support

Student appreciated the **focus on science in TBL and clinical reasoning in CBL:**

*You get to work a little bit harder in CBLs, in that smaller group with a doctor right there. So you get more out of it. But, also, they give you all the history and all the investigations in TBL, so you don’t really have to think about that. I think the things I learned from TBL is more like the science and theory side of it because we’re not made to think clinically. But in CBL, it kind of puts you in that, like, problem solving frame of mind.*

Participants felt there were **time efficiencies** associated with the CBL method that weren’t available in TBL:

*Very time effective. Everybody’s there, fully focused on the case, really getting down to it and getting it kind of done, and that was really great. CBL definitely makes sure that people don’t waste time and mess around, and distract the group - that definitely happens in TBL - wasted so much time.*

Students felt the CBL framework helped in knowledge retention because of the clinical context and additional problem solving compared to TBL:

*I think the advantage is I remember the cases, the CBL cases in my mind - I find that I can construct a picture of that patient and what disease and risk factors they had, and what investigations they had - much more easily in my mind than I can in previous TBLs because the information is, kind of, given to you in a TBL. Whereas working from first principles to try and figure out what the problem is, I think it helps you retain the information a little bit more. I’ll remember what I learnt in CBL. Well, I mean, I see a patient and, I recall the CBL stuff more than the TBL.*

#### Affective support

Students found in the CBL sessions students remained more engaged:

*There’s a lot of awkward silences in TBL when the doctor asks a question - not the case in CBL. You can’t hide as much - it pushes you to contribute more than the TBLs. In a smaller environment, you definitely stay focused and on task a lot better. You’re motivated to contribute in CBL because everyone’s kind of treated the same… you’ve all got the same amount of attention from the one clinician…in group discussions in TBL, it’s pretty much the same three people who answer the questions because everyone else is like a little bit nervous to like talk about, like say answers out loud in front of like 50 people - there’s a bit of fear amongst everyone in TBL.*

Students felt less comfortable to ask questions in TBL compared to CBL:

*In TBLs, there’s been times when I’ve wanted to ask a really stupid question, but then there’s been someone across the other side of the room who’s asked a really high level, detailed question - I don’t need to ask that in front of 60 people - I’ll find it out myself or ask someone else. Whereas I think this CBL environment is very welcoming of all questions, any level, any pitch, and it’s sort of not such a handbrake on the room discussion, it’s kind of just easy to get a quick answer - then move on and go forward as well, which I find much more welcoming and productive.*

Students suggested that the format of TBL promoted teamwork and development of leadership skills since it was more student directed than CBL:

*I find in TBL, because there’s less input from teachers, there is a bit more opportunity to learn communication skills and team leading skills, when you have people in your group that, maybe, aren’t as willing to contribute, it can be a skill to learn to engage everyone and try and deal with people that aren’t, maybe, working as hard…figure out on your own more. Whereas CBL, no one’s going to slack off because there’s a tutor right there. So you might not learn those communication and leadership skills as much.*

## Discussion

This study sought to explore students’ and facilitators’ perceptions of four piloted CBLs during Year 2 of the medical curriculum, across the Renal/Urology block, and draw some comparisons with their TBL experience to consider how the two approaches should co-exist within the curriculum. Findings indicate that students and facilitators found their experience in CBL to be positive, with many favourable aspects that built on and complemented their TBL experience. The learning environment was enriched by the CBL framework that allowed application of knowledge to solve clinical problems within the small groups with consistent facilitator guidance and feedback. Notably, compared to TBL, the CBL model provided increased opportunities for students to demonstrate their ability in clinical reasoning, and actively contribute to peer discussion and learning. Adaptation of teaching methods to meet student needs is required within the modern medical curriculum [17], and we now discuss our findings using the conceptual lens of Experience based learning (ExBL).

### Organisational support

Organisational support ensures that there are opportunities for active participation, and that these are aligned with learning and curriculum outcomes [14]. Students and facilitators commented that the CBL content was relevant to their clinical week, and well aligned with the curriculum. A strength of the CBL sessions was that both facilitators and students felt the format of the CBL mirrored that of a clinical consultation. They commented that this format built on their prior TBL experience which tended to emphasise more the basic science concepts of a particular case. It has been previously reported that the interactive components of the CBL process blend the cognitive and social aspects of learning to promote a deeper conceptual understanding of clinical concepts [18]. Students found the CBL sessions complemented this learning experience at an appropriate level for Year 2, where development of clinical reasoning skills is needed. However, students preferred the “flipped classroom” model of the TBL, and felt the CBL sessions would have benefited from the inclusion of specific pre-reading requirements. Students comments are aligned with the recent trend towards the “flipped classroom”, suggesting increased student satisfaction ‘in class’, engagement and learning outcomes [19]. Both students and facilitators commented that the LMS was lacking in CBLs, and inhibited their learning, with some patient data missing, and videos not working, which would require refinement.

### Pedagogic support

Pedagogic support indicates the support provided by tutors in practice-based learning [14]. Students felt the small group size of six students per group enhanced their learning experience, and achievement of their learning outcomes in comparison to the large class atmosphere of TBL. Facilitators and students noted that CBL provided time efficiencies not found in TBL, and that students were able to apply their knowledge systematically to a patient case. Use of authentic patient cases encourages discussion to emulate the complexities of clinical cognition, with the ability to expose students to complex clinical data and clinical care [4]. In CBL, discovery is encouraged in a format where both students and facilitators share responsibility for coming to closure on key learning points [20]. Facilitators indicated that students were much more forthcoming to ask questions in CBL compared to TBL, likely due to smaller group size with one facilitator present. Students’ ability to focus on key points of a clinical case encouraged a structured approach to clinical problem-solving. Literature suggests that active learning opportunities increase understanding of knowledge and assist in knowledge retention [21, 22].

### Affective support

Affective support occurs by provision of a learning environment that is inclusive [14]. A sense of trust is formed when students feel they are being treated as a member of a group with similar aims [23]. The learning environment afforded by the facilitators promoted supportive and constructive interactions among group members that fostered students’ development of clinical reasoning. Certainly clinical reasoning is a complex task, that is difficult to teach. It involves the integration and application of different types of knowledge and evidence, the critical analysis of patients’ symptoms, signs, laboratory results and imaging, and critical thinking to arrive at a diagnosis and management plan [24]. Literature suggests that active and engaging environments are required for the development of clinical reasoning [25, 26], and the small groups of six students in CBL with constant facilitator interaction enabled this process. Student learning was enhanced through open opportunities to ask questions and receive immediate feedback. Our findings are in line with a recent systematic review that suggests provision of immediate and specific feedback is a key component of CBL [4], and most students (87%) agreed that that they received useful and timely feedback from their facilitators.

### Resource implications for CBL and TBL

A benefit of TBL carried out on main university campus is the ability to standardise simultaneous implementation and facilitation of large classes across student cohorts. For example, for a cohort of 300 students, it is possible to run five TBL classes simultaneously, with 12 student teams in each classroom, requiring only three facilitators per class. There are obvious associated cost benefits to this. With CBL being implemented offsite in clinical schools, standardisation of implementation is reduced. The small group classes cannot be held simultaneously, more than twice the number of facilitators is required compared to TBL, with variability in their level of training, and a large number of small rooms are needed.

#### Study limitations

Our relatively small sample size and response rate may mean that our results may not be reflective of all students in the cohort, as we do not know how the views of these participants’ compare with the whole student cohort. We also acknowledge potential difficulties in implementing CBL across a large student cohort. Additionally, results may not be generalisable to other university settings. It is possible that students simply found the new method of teaching (CBL) to be novel, which may have made their responses more positive than if the study was carried out over a greater length of time.

## Conclusion

Both TBL and CBL emphasised the importance of activation of prior knowledge, application and integration of knowledge, and reasoning around problems. While the TBL model was integral in developing students’ knowledge and understanding of basic science concepts, the CBL model was integral in developing students’ clinical reasoning skills. Our findings indicate that delivery of a medical curriculum may be enhanced by optimising the instructional approaches of both teaching formats, with TBL being applied in earlier phases of the program where the focus is on basic science principles, and CBL becoming more relevant as students move towards clinical immersion. Students perceived the key benefits of their new CBL experience was the constant guidance provided by one facilitator to each small group, their capacity to focus discussion, and associated efficiencies in learning. Students’ learning processes were fostered appropriately by the organisational, pedagogic and affective support provided in both the TBL and CBL models, suggesting a need for increased vertical integration within the medical curriculum to align with the provision of early clinical experiences.

## Data Availability

Datasets supporting the conclusions of this article are included within the article. Additional data at the level of individual students is not available as per confidentiality agreements approved by the Human Research Ethics Committee, University of Sydney.

## Abbreviations

CBL: Case-based learning
TBL: Team-based learning
PBL: Problem based learning
SMS: Sydney Medical School
SMP: Sydney Medical Program
ExBL: Experience based learning
